# The Role of Catalase C262T Gene Polymorphism in the Susceptibility and Survival of Cancers

**DOI:** 10.1038/srep26973

**Published:** 2016-05-26

**Authors:** Cheng-Di Wang, Yan Sun, Nan Chen, Lin Huang, Jing-Wen Huang, Min Zhu, Ting Wang, Yu-Lin Ji

**Affiliations:** 1 Department of Respiratory and Critical Care Medicine, West China Medical School/West China Hospital, Sichuan University, Chengdu, Sichuan Province 610041, PR China; 2 West China School of Medicine/West China Hospital, Sichuan University, Chengdu, Sichuan 610041, PR China

## Abstract

Catalase (CAT), one antioxidant enzyme, may provide resistance against many diseases. Many previous studies reported predictive and prognostic values of CAT C262T polymorphism in cancers, with divergent results. This study aimed to summarize the overall relationships between CAT C262T polymorphism and cancer risk or survival. A total of 27 eligible publications were included in susceptibility analysis, while 8 publications contained survival outcomes. The results revealed significant relationship between CAT C262T polymorphism and cancer risk(TT + CT vs CC: OR = 1.05, 95%CI = 1.00–1.10, P = 0.036), subgroup analyses indicated the CAT C262T polymorphism was significantly correlated with an increased risk for prostate cancer (TT vs CC + CT: OR = 1.43, 95%CI = 1.20–1.70, P < 0.001) and increased risk among Caucasians (TT vs CC + CT: OR = 1.19, 95%CI = 1.09–1.31, P < 0.001), while no associations between the polymorphism and Asian or mixed population were established. In the survival analysis, no interactions were identified between this polymorphism and cancer survival (TT + CT vs CC: HR = 1.37, 95%CI = 0.70–2.70, P = 0.36). In conclusion, the CAT C262T polymorphismmay be a candidate markerfor cancer risk with type-specific and population-specific effects but not a fine prognostic factor for cancer survival.

The molecular mechanisms of carcinogenesis have not been wellunderstood, but growing studies have reported that oxidative stress played a significant role in the progression of many diseases, including cancers^1^. Oxidative stress could contribute to imbalance between the reactive oxygen species (ROS) and antioxidant defense system^2^. When present at high and/or sustained level, ROS may induce severe DNA damage and chromosomal aberrations^3^,^4^,^5^, which may be followed by abnormal expression of proto-oncogenes and tumor suppressor genes. However, antioxidant defense system could prevent or combat the negative effects caused by ROS, including myeloperoxidase (MPO), glutathione peroxidase (GPX), catalase (CAT), and mitochondrial manganese superoxide dismutase (MnSOD)^6^,^7^,^8^.

Catalase is an important endogenous antioxidant enzyme thatcatalyzes hydrogen peroxide into oxygen and water, thus neutralizing the deleterious effects of ROS^9^. The CAT gene, which is located on chromosome11p13, consists of 12 introns and 13 exons^10^. There are several single nucleotide polymorphisms (SNPs) identified in the CAT gene, of which the rs1001179 polymorphism (C262T) was the most extensively studied^11^,^12^. The CAT C262T polymorphism is encoded on the promoter region, influencing transcriptional and splicing regulation^13^. In comparison with the variant C allele, the variant T allele of the CAT C262T polymorphism has been reported to indicate lower enzyme activity, thus raising the levels of ROS and might lead to cancer development or progression^14^. Recently, a series of studies has demonstrated the associations between the CAT C262T polymorphism and risk for multiple cancers, such as breast cancer^15^, prostate cancer^16^, hepatocellular carcinoma^11^, chronic myeloid leukemia^17^, etc. So far, some studies have indicated the CAT C262T polymorphismcould increase prostate cancer risk^6^,^16^,^18^. However, the final results were not consistent or conclusive. In terms of survival, no studies confirmed whether the CAT C262T polymorphism could be a prognostic factor of cancer patients. Here, we conducted this updated meta-analysis to comprehensively estimate the relationships between the CAT C262T polymorphism and susceptibility or survival of cancers.

## Results

### Eligible studies

**Figure 1:**
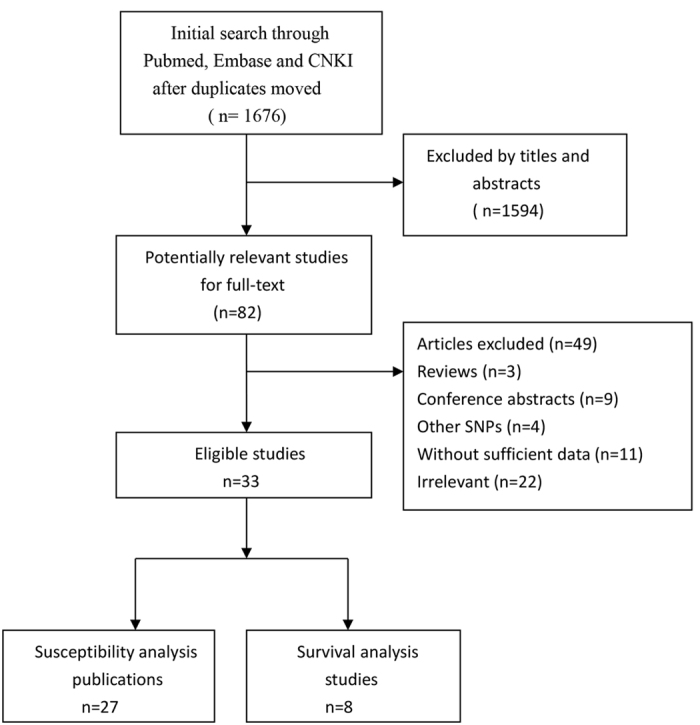
Flow chart of study inclusion and exclusion in this meta-analysis.

The initial search yielded 1676 articlesthrough the databases of Pubmed, Embase and China National Knowledge Infrastructure (CNKI). After screening the titles and abstracts, 82 potentially relevant articles were retrieved for the full-text. 49 articles were excluded: 3 were reviews; 9 were conference abstracts; 4 were related to other SNPs of the CAT gene; 11 did not report extractable data; 22 were irrelevant papers. Finally, a total of 33 articles^6^,^7^,^8^,^11^,^12^,^15^,^16^,^17^,^18^,^19^,^20^,^21^,^22^,^23^,^24^,^25^,^26^,^27^,^28^,^29^,^30^,^31^,^32^,^33^,^34^,^35^,^36^,^37^,^38^,^39^,^40^,^41^,^42^ published from 2005 to 2015met the inclusion criteria and were included in our meta-analysis. There were 27 publications^6^,^7^,^8^,^11^,^12^,^15^,^16^,^17^,^18^,^19^,^21^,^23^,^24^,^25^,^26^,^27^,^28^,^29^,^30^,^31^,^33^,^34^,^35^,^37^,^38^,^39^,^40^ regarding susceptibility analysis, which involved 35 case-control or cohort studies with 15531 cancer patients and 41816 controls, while 8 publications^6^,^20^,^22^,^29^,^32^,^36^,^41^,^42^ contained the survival data. The search process was presented in Fig. 1 and the clinical characteristics of the studies or other relevant information were listed in Table 1.

### C262T polymorphism and susceptibility to cancer

**Figure 2:**
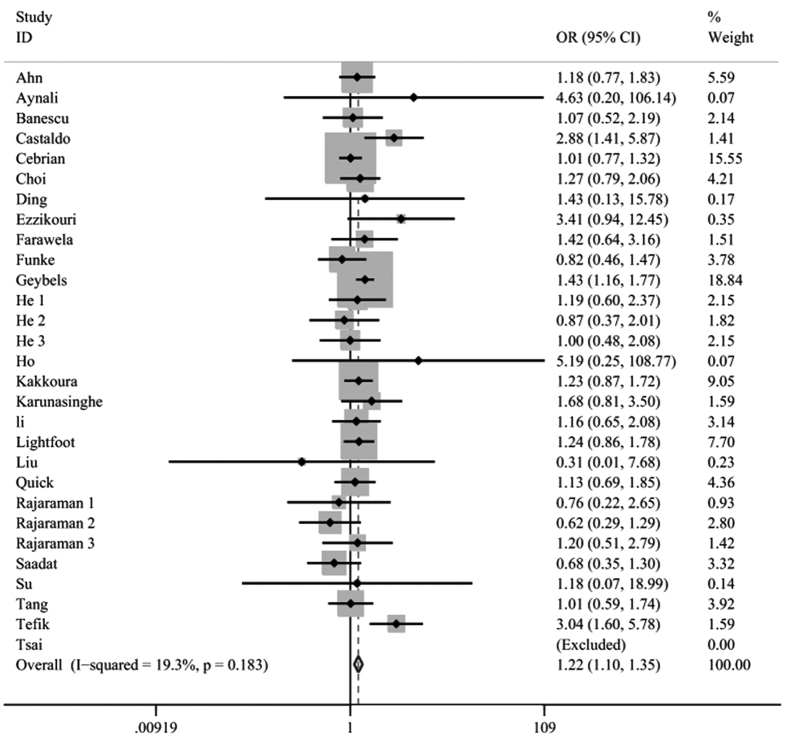
Forest plot for the association between the CAT C262T polymorphism and cancer risk (TT vs CC). Significant association was observed between the CAT C262T polymorphism and cancer susceptibility.

The meta-analysis of the 27 articles^6^,^7^,^8^,^11^,^12^,^15^,^16^,^17^,^18^,^19^,^21^,^23^,^24^,^25^,^26^,^27^,^28^,^29^,^30^,^31^,^33^,^34^,^35^,^37^,^38^,^39^,^40^ with 35 case-control or cohort studies suggested there was a positive correlation between the CAT C262T polymorphism and cancer risk (TT + CT vs CC: OR = 1.05, 95%CI = 1.00–1.10, P = 0.036; TT vs CT + CC: OR = 1.18, 95%CI = 1.08–1.29, P < 0.001; TT vs CC: OR = 1.22, 95%CI = 1.10–1.35, P < 0.001; T vs C: OR = 1.07, 95%CI = 1.03–1.11, P = 0.001 Fig. 2). In the studies which were not derived from the Hardy-Weinberg equilibrium (HWE), the pooled ORs also showed the significance of CAT C262T polymorphism in susceptibility to cancers (TT vs CT + CC: OR = 1.15, 95%CI = 1.02–1.28, P = 0.019; TT vs CC: OR = 1.14, 95%CI = 1.02–1.28, P = 0.026). Furthermore, a subgroup analysis was also performed stratified by cancer types and ethnicity. There was a significant association between CAT C262T polymorphism and the development of prostate cancer^6^,^7^,^16^,^18^,^25^,^26^ (TT vs CT + CC: OR = 1.43, 95%CI = 1.20–1.70, P < 0.001; TT vs CC: OR = 1.52, 95%CI = 1.27–1.81, P < 0.001; CT vs CC: OR = 1.15, 95%CI = 1.05–1.26, P = 0.002; T vs C: OR = 1.21, 95%CI = 1.05–1.40, P = 0.01). The association between the polymorphism of the CAT C262T gene and increased skin cancer risk was also confirmed^30^ (CT + TTvs CC: OR = 1.19, 95%CI = 1.00–1.41, P = 0.04; CT vs CC: OR = 1.21,95%CI = 1.02–1.44, P = 0.03). Meanwhile, the CAT C262T polymorphism retained its high position for predicting the susceptibility to cervical cancer^12^ (TT vs CT + CC: OR = 2.85, 95%CI = 1.44–5.65, P = 0.003; TT vs CC: OR = 2.88, 95%CI = 1.41–5.87, P= 0.004; T vs C: OR = 1.96, 95%CI = 1.31–2.93, P = 0.001). However, no evidence of statistical significance could be detected in other cancer types. In terms of subgroup analysis by ethnicity (Caucasian, Asian and Mixed), the assessment of the results revealed that the CAT C262T polymorphism was associated with cancer risk in Caucasians (TT vs CT + CC: OR = 1.19, 95%CI = 1.09–1.31 P  < 0.001; TT vs CC: OR = 1.24, 95%CI = 1.12–1.38, P < 0.001; T vs C: OR = 1.08, 95%CI = 1.01–1.16, P = 0.02). No relationship could be found in Asian or mixed population. The pooled results were shown in Table 2.

### C262T polymorphism and cancer survival

The meta-analysis included 8 studies investigating CAT C262T polymorphism and cancer survival^6^,^20^,^22^,^29^,^32^,^36^,^41^,^42^. No overall survival (OS) difference was detected between patients with CT/TT genotypes and those with CC genotype (HR = 1.37, 95%CI = 0.70–2.70, P = 0.36), or between patients with TT genotype and allele C carrier (HR = 0.90, 95%CI = 0.44–1.83, P = 0.77). Furthermore, when compared to CC genotype, CT or TT genotype didn’t suggest poorer OS (HR = 1.07, 95%CI = 0.95–1.20, P = 0.29; HR = 1.04, 95%CI = 0.81–1.34, P = 0.74, respectively). In addition, cancer patients with T allele showed similar survival compared to those with C allele (HR = 1.07, 95%CI = 0.97–1.18, P = 0.21). The main results were summarized in Table 3.

### Publication bias and sensitivity analysis

**Figure 3:**
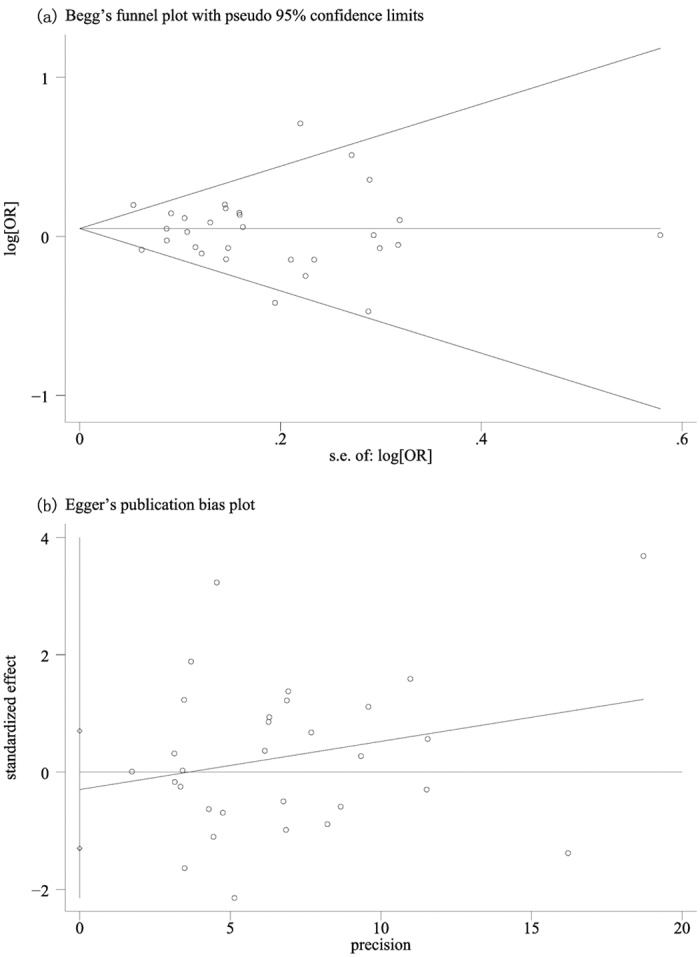
Begg’s funnel plot and Egger’s on publication bias for included studies on the association of the CAT C262T polymorphism and cancer risk (TT vs CC). The funnel plot seemed symmetrical, indicating absence of publication bias.

We didn’t detect any significant publication bias by Begg’ test (Pr > |z| = 0.775 Fig. 3a) or Egger’ test (P > |t| = 0.548 Fig. 3b), which indicated the reliability of our meta-analysis. Furthermore, no significant change was detected when we sequentially dropped out each included study and thus the results of our study were stable.

## Discussion

ROS are naturally generated fromaerobic metabolism^3^. The human body develops a sophisticated set of antioxidant molecules to prevent the toxic accumulation of these species^43^. CAT belongs to the antioxidant molecules and is present in all aerobic cells while the highest levels of the enzyme are found in the liver, kidneyand erythrocytes^44^. CAT is a heme enzyme that plays a very important role in avoiding hydrogen peroxide concentration by converting H_2_O_2_ into H_2_O and O_2_, and protects cells from detrimental effects of oxidative stress^45^. Allelic variants of CAT gene may contribute to lower CAT enzymatic activity and higher sensitivity to ROS, and alter ROS detoxification and increase oxidative stress, thereby implicating oxidative DNA damage and modulating disease risk^46^. 245 CAT SNPs have been identified, with most studies investigating the relationships between multiple diseases and rs1001179, a C > T substitution at position −262 from the transcription start site^44^. Previous studies indicated thatCAT C262T gene polymorphism had an influence on transcription factors binding thus altering the basal transcription and consequent expression of this enzyme and hence influenced the oxidative status of cells and its microenvironment^25^,^26^. Consequently, this polymorphism was believed to play a key role in the pathogenesis of cancer^25^,^26^. The growing studies investigated the relation of CAT C262T gene polymorphism to breast cancer, lung cancer, diabetic neuropathy, non-Hodgkin lymphoma, liver cancer and colorectal cancer^43^, however, these results did not reach an agreement. A meta-analysis is a useful strategy because it potentially investigates a large number of individuals and could evaluate the effect of a genetic factor oncancer risk. We performed the current meta-analysis to combine the eligible studies and data to precisely estimate the role of CAT C262T polymorphism in the susceptibility and survival of cancers.

The present meta-analysis, including 15531 cancer patients and 41816 controls from 35 case–control or cohort studies, investigated the association between the CAT C262T polymorphism and cancer risk. Based on current accessible evidences, the individuals who carry the TT homozygote have 17% increased risk of cancer compared with the C allele carriers, revealing that the CAT C262T gene polymorphism may be a risk factor for cancer^47^. For tumor origin could influence the results from meta analysis, we performed subgroup analyses by cancer type. However, we did not find any positive relationship in the studies of breast cancer, head and neck cancer, hematological malignancies, digestive system cancer or brain cancer. Interestingly, the significant association between the CAT C262T gene and prostate cancer^6^,^7^,^16^,^18^,^25^,^26^ was the opposite in most genetic models. The relationships between the CAT C262T gene and skin cancer^31^ or cervical cancer^12^ were opposite in part genetic models. Meanwhile, in the stratified analysis by ethnicity, significantly elevated cancer risks were indicated in Caucasian group but not in Asian population. The underlying genetic backgrounds and/or environmental and social factors may account for the ethnic discrepancy.

It is worth mentioning that the current study was the first meta-analysis to investigate the survival outcomes. While the TT genotype was associated with increased cancer risk especially in prostate cancer and Caucasian population, however, neither of TT or CT genotype contributed to poorer survival of cancer patients. These results indicated that CAT C262T polymorphism might only influence susceptibility to cancer instead of cancer prognosis. In addition, the association between C262T polymorphism and treatment efficiency such as chemotherapy and radiotherapy remained unclear and those data were insufficient to reach a pooled result. Further studies could focus on the role of CAT C262T polymorphism on treatment strategy. The exact mechanisms of the C262T polymorphism on cancer development and progression were warranted to investigate in future.

In interpreting the current results, several limitations of the meta-analysis should be addressed. Only if literatures that were indexed by the selected databases were included for the current study, and some relevant published studies or unpublished studies with null results were missed or ongoing studies were not sought, which might have influenced our results. Secondly, the numbers of published studies were not large to identify possible associations, especially in survival analysis. Thirdly, part studies investigated several cases with the same control, which might reduce the statistical power to identify possible associations. Fourthly, lacking the original data of the reviewed studies limited our further evaluation of the potential interaction. However, our current study also had some merits. On one hand, over 30 case-control or cohort studies from different publications significantly increased statistical power of the analyses. On the other hand, on the basis of our studies, we find a novel mechanism to predict cancer risk. In addition, the current study is the first to investigate the survival outcomes.

To sum up, the results from the current study suggest that the CAT C262T polymorphism may contribute to genetic susceptibility to cancer, supporting the hypothesis that the polymorphism serves as a potential susceptibility tumor marker. However the CAT C262T polymorphismmay not be a fine prognostic factor for cancer survival. Further well-designed, multicenter epidemiological studies including a wider spectrum of subjects should be performed to investigate the role of this functional polymorphism in other populations and biological mechanism of CAT C262T polymorphism, which should lead to better, comprehensive interpretation of the association between the CAT C262T polymorphism and cancer risk.

## Methods

### Identification and Eligibility of Relevant Studies

Two investigators performed a comprehensive and systematic search through the databases of Pubmed, Embase and CNKI for relevant studies with the following terms: “catalase” or “CAT”, “polymorphism” or “variant” or “mutation”, and “cancer” or “carcinoma” or “malignancy” (Last search update December 2015). The publication language and publication date were not restricted in our search. Some potential publications were obtained from a manual search of the references of retrieved articles.

The inclusion criteria were: (1) case-control studies or cohort studies; (2) evaluating the associations between the CAT C262T polymorphism and cancer risk or survival outcomes; (3) detailed data on genotype frequency of the CAT C262T for calculating the odds ratios (ORs), available hazards ratios (HRs) and 95% confidence intervals (95%CIs). The exclusion criteria were: (1) reviews, conference abstracts, case reports, animal studies or editorials; (2)without available genotype frequency of the CAT C262T; (3) when the same or overlapped population and duplicated studies were met, only the most recent studies with sufficient information were included.

### Data extraction

Two investigators extracted data independently and consensus on all the items was reached after discussion. The main information included the first author, publication year, country, ethnicity, source of controls, sample, quality control, quality health, cancer type, number of cases and controls, genotype distributions of cases and controls, genotyping method, HWE of the control groups, and HR with 95%CI of this polymorphism in survival analysis.

### Statistical Analysis

All statistical analyses were conducted with STATA 12.0 (Stata Corp, College Station, TX, USA). The statistical heterogeneity among the studies was calculated by the I^2^ statistics. If I^2^ > 50%, the random-effects model was applied to analysis; otherwise, the fixed-effects model was adopted^48^,^49^. ORs with 95% CIs were used to stabilize risk estimates, while HRs with 95% CIs were required to predict whether the CAT C262T gene polymorphism had influence on OS of cancer patients. The following genetic models were used to evaluate the susceptibility: dominant model (TT + CT vs CC), recessive model (TT vs CT + CC), homozygote model (TT vs CC), heterozygote model (CT vs CC), and allelic contrast model (T vs C). We also performed the subgroup analyses based on cancer type and ethnicity. The significance of the pooled OR was assessed by Z test and the statistically significant outcome was defined as P < 0.05. HWE was evaluated by the chi-square test in control groups for each study, where P < 0.05 was considered significant^50^. Both Egger’s and Begg’s tests were used to evaluate the publication bias^51^. Sensitivity analysis, which aimed to identify whether the heterogeneity across these studies was from one individual study, was also performed to ensure the reliability of the results.

## Additional Information

**How to cite this article**: Wang, C.-D. *et al.* The Role of Catalase C-262T Gene Polymorphism in the Susceptibility and Survival of Cancers. *Sci. Rep.* **6**, 26973; doi: 10.1038/srep26973 (2016).

## Supplementary Material

Supplementary Information

## Figures and Tables

**Table 1:** Baseline characteristics of eligible studies (N = 33).

First Author	#^*^	Year	Country	Ethnicity	Source of Controls	Quality Control	Cancer Type	Case/Control	Genotyping Method	HWE
Ahn^19^		2005	USA	Caucasian	PB	Yes	Breast cancer	1008/1056	MALDI-TOF	Yes
Ambrosone^20^		2005	USA	Mixed	PB	NA	Breast cancer	279/NA	MALDI-TOF	NA
Aynali^21^		2013	Turkey	Caucasian	HB	NA	Laryngeal cancer	25/23	PCR	Yes
Banescu^17^		2014	Romania	Caucasian	HB	NA	CML	168/321	PCR-RFLP	Yes
Belotte^22^		2015	USA	Mixed	NA	NA	Ovarian cancer	NA	TaqMan	NA
Bhatti^23^	1	2009	USA	Caucasian	HB	Yes	Glioma	362/494	TaqMan	NA
Bhatti^23^	2	2009	USA	Caucasian	HB	Yes	Glioblastoma multiforme	176/494	TaqMan	NA
Bhatti^23^	3	2009	USA	Caucasian	HB	Yes	Meningioma	134/494	TaqMan	NA
Castaldo^12^		2015	Portugal	Caucasian	HB	NA	Cervical cancer	120/107	PCR	No
Cebrian^24^		2006	UK	Caucasian	PB	Yes	Breast cancer	2171/2262	TaqMan	Yes
Cheng^25^		2011	USA	mixed	PB	NA	Prostate cancer	150/761	PCR	NA
Choi^7^		2007	USA	Mixed	PB	Yes	Prostate cancer	508/1403	MALDI-TOF	Yes
Ding^26^		2012	China	Asian	PB	NA	Prostate cancer	1417/1008	HapMap	Yes
Ezzikouri^27^		2010	France	Caucasian	HB	Yes	Hepatocellular carcinoma	96/222	PCR-RFLP	Yes
Farawela^28^		2012	Egypt	Caucasian	HB	Yes	NHL	100/100	PCR-RFLP	Yes
Funke^29^		2009	Germany	Caucasian	PB	Yes	Colorectal Cancer	632/605	Pyrosequencing Technology	Yes
Geybels^6^		2014	Netherland	Caucasian	PB	Yes	Prostate cancer	1527/25184	PCR	No
He^30^	1	2010	USA	Caucasian	PB	NA	BCC	270/796	TaqMan	Yes
He^30^	2	2010	USA	Caucasian	PB	NA	Melanoma	211/796	TaqMan	Yes
He^30^	3	2010	USA	Caucasian	PB	NA	SCC	266/796	TaqMan	Yes
Ho^31^		2006	China	Asian	HB	NA	Lung cancer	230/240	PCR-RFLP	Yes
Kakkoura^15^		2015	Cyprus	Caucasian	PB	Yes	Breast cancer	1057/1141	TaqMan	Yes
Karunasinghe^16^		2012	New Zealand	Caucasian	HB	NA	Prostate cancer	258/434	TaqMan	Yes
Koistinen^32^		2006	Finland	Caucasian	NA	Yes	AML	89/NA	PCR	NA
Li^33^		2009	USA	Caucasian	PB	Yes	Breast cancer	497/493	TaqMan	Yes
Lightfoot^34^		2006	USA/UK	Caucasian	PB	NA	NHL	928/1446	TaqMan	Yes
Liu^35^		2015	China	Asian	PB	Yes	Hepatocellular carcinoma	266/248	PCR-RFLP	Yes
Nahon^36^		2009	France	Caucasian	NA	NA	Hepatocellular carcinoma	190/NA	PCR	NA
Quick^37^	1	2008	USA	Mixed	PB	Yes	Breast cancer	57/108	MALDI-TOF	Yes
Quick^37^	2	2008	USA	Caucasian	PB	Yes	Breast cancer	569/974	MALDI-TOF	Yes
Rajaraman^8^	1	2008	USA	Mixed	HB	Yes	Acoustic neuroma	69/494	TaqMan	Yes
Rajaraman^8^	2	2008	USA	Mixed	HB	Yes	Glioma	362/494	TaqMan	Yes
Rajaraman^8^	3	2008	USA	Mixed	HB	Yes	Meningioma	134/494	TaqMan	Yes
Saadat^38^		2015	Iran	Caucasian	PB	NA	Breast cancer	407/395	PCR	Yes
Su^11^		2015	China	Asian	HB	Yes	Hepatocellular carcinoma	400/480	PCR-RFLP	Yes
Tang^39^		2010	USA	Mixed	HB	NA	Pancreatic cancer	551/602	TaqMan	Yes
Tefik^18^		2013	Turkey	Caucasian	HB	NA	Prostate cancer	155/195	PCR	Yes
Tsai^40^		2012	China	Asian	HB	Yes	Breast cancer	260/224	PCR	Yes
Ulder^41^		2007	England	Caucasian	PB	Yes	Breast cancer	NA	TaqMan	NA
Van Blarigan^42^		2014	USA	Caucasian	PB	NA	Prostate cancer	NA	MALDI-TOF	NA

*Number of data separately reported by articles.

HWE: Hardy-Weinberg equilibrium; MALDI-TOF: Matrix-Assisted Laser Desorption/ Ionization Time of Flight Mass Spectrometry; PCR: polymerase chain reaction; PCR-RFLP: polymerase chain reaction-restriction fragment length polymorphism; PB: population-based; HB: hospital-based; NA: not available. CML: Chronic myeloid leukemia; NHL: non-Hodgkin lymphoma; BCC: Basal cell carcinoma; SCC: Squamous cell carcinoma; AML: Acute myeloid leukemia.

**Table 2:** The results of evidence synthesis of susceptibility analysis.

Variables	Dominant model (TT + CT vs CC)			Recessive model (TT vs CT + CC)			Homozygote model (TT vs CC)			Heterozygote model (CT vs CC)			Allel contrast model (T vs C)		
	OR(95%CI)	P	I^2^ (%)	OR(95%CI)	P	I^2^ (%)	OR(95%CI)	P	I^2^ (%)	OR(95%CI)	P	I^2^ (%)	OR(95%CI)	P	I^2^ (%)
All	1.05(1.00–1.10)	0.036	39.80	1.18(1.08–1.29)	<0.001	2.20	1.22(1.10–1.35)	<0.001	19.30	1.03(0.98–1.08)	0.23	28.90	1.07(1.03–1.11)	0.001	47.60
By cancer type															
Breast cancer	1.02(0.95–1.10)	0.58	30.40	1.08(0.92–1.27)	0.36	0.00	1.08(0.92–1.27)	0.37	0.00	1.01(0.94–1.09)	0.75	25.40	1.03(0.97–1.09)	0.42	26.70
Hematological malignancies	0.92(0.79–1.07)	0.30	46.20	1.30(0.98–1.74)	0.07	0.00	1.23(0.91–1.66)	0.18	0.00	0.82(0.60–1.13)	0.23	51.90	0.99(0.88–1.12)	0.92	26.50
Brain cancer	0.86(0.69–1.06)	0.16	0.00	1.02(0.85–1.23)	0.80	0.00	0.80(0.48–1.34)	0.40	0.00	0.86(0.69–1.08)	0.2	2.30	0.88(0.73–1.05)	0.17	0.00
Prostate cancer	1.15(0.98–1.36)	0.09	58.10	1.43(1.20–1.70)	<0.001	0.00	1.52(1.27–1.81)	<0.001	26.20	1.15(1.05–1.26)	0.002	22.30	1.21(1.05–1.40)	0.01	61.90
Digestive system cancer	0.92(0.79–1.06)	0.24	0.00	1.05(0.73–1.50)	0.81	15.10	1.01(0.70–1.46)	0.95	9.40	0.91(0.80–1.05)	0.19	0.00	0.94(0.83–1.07)	0.36	0.00
Skin cancer	1.19(1.00–1.41)	0.04	0.00	0.96(0.63–1.47)	0.86	0.00	1.03(0.67–1.58)	0.90	0.00	1.21(1.02–1.44)	0.03	0.00	1.13(0.98–1.30)	0.10	0.00
By ethnicity															
Caucasian	1.06(0.98–1.15)	0.13	50.20	1.19(1.09–1.31)	<0.001	14.10	1.24(1.12–1.38)	<0.001	31.00	1.04(0.98–1.09)	0.18	39.80	1.08(1.01–1.16)	0.02	58.00
Asian	1.04(0.85–1.28)	0.72	NA	1.41(0.40–5.00)	0.60	0.00	1.40(0.39–4.98)	0.60	0.00	1.03(0.84–1.27)	0.78	0.00	1.05(0.86–1.28)	0.66	0.00
Mixed	0.91(0.72–1.16)	0.45	52.40	0.94(0.65–1.35)	0.73	0.00	0.89(0.62–1.29)	0.55	0.00	0.96(0.70–1.31)	0.78	64.90	0.93(0.81–1.06)	0.27	49.10
By HWE															
Yes	1.01(0.96–1.07)	0.58	0.13	1.15(1.02–1.28)	0.02	0.70	1.14(1.02–1.28)	0.03	0.50	1.00(0.95–1.05)	0.93	0.20	1.03(0.99–1.07)	0.19	0.12
No	1.23(1.11–1.37)	<0.001	0.26	1.82(0.88–3.75)	0.10	0.04	1.86(0.96–3.63)	0.07	0.06	1.18(1.06–1.32)	0.003	0.70	1.47(0.91–2.38)	0.11	0.02

P: P-value of Z-test to evaluate the significance of the ORs; NA: not available.

**Table 3:** The results of evidence synthesis of overall survival analysis.

Model	Variables	N^*^	HR(95%CI)	P	I^2^(%)
Dominant model	CC	3	Reference	0.358	66.7%
	CT/TT		1.37(0.70–2.70)		
Recessive model	CC/CT	2	Reference	0.77	0%
	TT		0.90(0.44–1.83)		
Homozygote model	CC	6	Reference	0.744	17.1%
	TT		1.04(0.81–1.34)		
Heterozygote model	CC	6	Reference	0.29	0%
	CT		1.07(0.95–1.20)		
Allelic model	C	4	Reference	0.21	9.6%
	T		1.07(0.97–1.18)		

*Number of studies in analysis.
