# Supplementary material for: The Role of Catalase C262T Gene Polymorphism in the Susceptibility and Survival of Cancers
Source: Sci Rep. 2016 May 26;6:26973. doi: 10.1038/srep26973 (PMC4880922; doi:10.1038/srep26973)
Supplement: Supplementary Information [file srep26973-s1.pdf]

The Role of Catalase C-262T Gene Polymorphism in the Susceptibility and Survival of Cancers

Running head: Catalase C-262T polymorphism and cancer

Cheng-Di Wang[1,#], Yan Sun[2,#], Nan Chen[2,#], Lin Huang[2], Jing-Wen Huang[2], Min Zhu[1], Ting Wang[1], Yu-Lin Ji[1*]

[1]*Department of Respiratory and Critical Care Medicine, West China Medical School/West China Hospital, Sichuan University, Chengdu,Sichuan Province 610041, PR China*

[2]*West China School of Medicine/West China Hospital, Sichuan University, Chengdu, Sichuan 610041, PR China*

***Corresponding author：** Yu-Lin Ji

Address: Department of Respiratory and Critical Care Medicine, West China Medical School/West China Hospital,Sichuan University,No. 37 Guo Xue Alley, Chengdu, Sichuan Province. **Post Code:** 610041, PR China. **Tel:** +86 28 85422571; **Fax:** +86 28 85422571; **E-mail:**jiyulinscu@sina.cn

[#]These authors contributed equally to this work.

Supplementary Table. 1 Genotype frequency distribution of CAT C-262T gene polymorphism

First author	#*	Ethnicity	Cancer Type	Case			Control			HWE
				CC	CT	TT	CC	CT	TT	
Ahn		Caucasian	Breast cancer	614	349	45	679	335	42	Yes
Aynali		Caucasian	Laryngeal cancer	13	10	2	12	11	0	Yes
Banescu		Caucasian	CML	105	49	14	168	132	21	Yes
Bhatti	1	Caucasian	Glioblastoma multiforme	132		186	186		249	NA
Bhatti	2	Caucasian	Meningioma	65		85	186		249	NA
Bhatti	3	Caucasian	Glioblastoma multiforme	46		72	186		249	NA
Castaldo		Caucasian	Cervical cancer	58	25	36	65	27	14	No
Cebrian		Caucasian	Breast cancer	1351	707	113	1362	787	113	Yes
Cheng		Mixed	Prostate cancer	69	33		450	276		NA
Choi	1	Caucasian	Prostate cancer	281	157	25	732	445	56	Yes
Choi	2	African-American	Prostate cancer	24	3	0	109	11	0	Yes
Ding		Asian	Prostate cancer	1316	99	2	940	67	1	Yes
Ezzikouri		Caucasian	Hepatocellular carcinoma	76	14	6	173	45	4	Yes
Farawela		Caucasian	NHL	26	49	25	28	53	19	Yes
Funke		Caucasian	Colorectal Cancer	374	235	23	348	231	26	Yes
Geybels		Caucasian	Prostate cancer	887	539	103	15794	8108	1282	No
He	1	Caucasian	BCC	161	97	12	512	252	32	Yes
He	2	Caucasian	Melanoma	129	75	7	512	252	32	Yes
He	3	Caucasian	SCC	160	96	10	512	252	32	Yes
Ho		Asian	Lung cancer	209	19	2	217	23	0	Yes
Kakkoura		Caucasian	Breast cancer	609	369	79	671	399	71	Yes
Karunasinghe		Caucasian	Prostate cancer	144	99	15	258	160	16	Yes
Li		Caucasian	Breast cancer	295	176	26	303	167	23	Yes

Lightfoot		Caucasian	NHL	554	298	57	867	498	72	Yes
Liu		Asian	Hepatocellular carcinoma	239	27	0	223	24	1	Yes
Quick	1	Mixed	Breast cancer	34	13	0	97	10	1	Yes
Quick	2	Caucasian	Breast cancer	345	197	27	598	333	43	Yes
Rajaraman	1	Mixed	Acousticneuroma	43	17	3	251	164	23	Yes
Rajaraman	2	Mixed	Glioma	195	124	11	251	164	23	Yes
Rajaraman	3	Mixed	Meningioma	73	39	8	251	164	23	Yes
Saadat		Caucasian	Breast cancer	261	129	17	240	132	23	Yes
Su		Asian	Hepatocellular carcinoma	365	34	1	432	47	1	Yes
Tang		Mixed	Pancreatic cancer	349	174	28	366	207	29	Yes
Tefik		Caucasian	Prostate cancer	58	64	33	107	68	20	Yes
Tsai		Asian	Breast cancer	225	35	0	202	22	0	Yes

* number of data separately reported by articles.

HWE: Hardy-Weinberg equilibrium; CML: Chronic myeloid leukemia; NHL=non-Hodgkin lymphoma; BCC: Basal cell carcinoma; SCC: Squamous cell carcinoma.

Supplementary Table.2 The extracted data of survival analysis

Model	Studies	Year	Ethnicity	Cancer Type	HR(95%CI)
Dominant model	Belotte	2015	Mixed	Ovarian Cancer	3.69(1.15-11.84)
	Koistinen	2006	Caucasian	AML	0.80(0.50-1.50)
	Nahon	2009	Caucasian	Hepatocellular Carcinoma	1.40(0.80-2.20)
Recessive model	Ambrosone	2005	Caucasian	Breast Cancer	0.50(0.12-2.00)
	Koistinen	2006	Caucasian	AML	1.10(0.50-2.60)
Homozygote model	Ambrosone	2005	Caucasian	Breast Cancer	0.49(0.12-2.02)
	Funke	2009	Caucasian	Rectal Cancer	0.58(0.13-2.71)
	Geybels	2014	Caucasian	Prostate Cancer	1.38(0.97-1.97)
	Van Blarigan	2014	Caucasian	Prostate Cancer	0.66(0.32-1.35)
	Koistinen	2006	Caucasian	AML	0.90(0.40-2.40)
	Udler	2007	Caucasian	Breast Cancer	0.90(0.53-1.52)
Heterozygote model	Ambrosone	2005	Caucasian	Breast Cancer	1.00(0.62-1.61)
	Funke	2009	Caucasian	Rectal Cancer	1.37(0.65-2.87)
	Geybels	2014	Caucasian	Prostate Cancer	1.15(0.96-1.36)
	Van Blarigan	2014	Caucasian	Prostate Cancer	1.10(0.83-1.47)
	Koistinen	2006	Caucasian	AML	0.70(0.40-1.30)
	Udler	2007	Caucasian	Breast Cancer	0.96(0.76-1.22)
Allelic model	Funke	2009	Caucasian	Rectal Cancer	1.16(0.58-2.30)
	Geybels	2014	Caucasian	Prostate Cancer	1.16(1.01-1.33)
	Van Blarigan	2014	Caucasian	Prostate Cancer	0.97(0.77-1.21)
	Udler	2007	Caucasian	Breast Cancer	0.96(0.79-1.16)

AML: Acute myeloid leukemia
